# p-Type Conjugated Polymers Containing Electron-Deficient Pentacyclic Azepinedione

**DOI:** 10.1021/acs.macromol.3c00843

**Published:** 2023-07-26

**Authors:** Qiao He, Jessica Shaw, Yuliar Firdaus, Xiantao Hu, Bowen Ding, Adam V. Marsh, Alexandre S. Dumon, Yang Han, Zhuping Fei, Thomas D. Anthopoulos, Christopher R. McNeill, Martin Heeney

**Affiliations:** † Department of Chemistry and Centre for Processable Electronics, Imperial College London, White City Campus, London W12 0BZ, U.K.; ‡ KAUST Solar Center (KSC), Physical Science and Engineering Division (PSE), King Abdullah University of Science and Technology (KAUST), Thuwal 23955-6900, Saudi Arabia; § School of Materials Science & Engineering, Tianjin Key Laboratory of Molecular Optoelectronic Sciences, Collaborative Innovation Center of Chemical Science and Engineering (Tianjin), Tianjin University, Tianjin 300072, China; ∥ Institute of Molecular Plus, Department of Chemistry, Tianjin Key Laboratory of Molecular Optoelectronic Science, Tianjin University, Tianjin 300072, China; ⊥ Department of Materials Science and Engineering, Monash University, Clayton, Victoria 3800, Australia; # Department of Chemistry and Centre for Processable Electronics, Imperial College London, London W12 0BZ, U.K.; ¶ Research Center for Electronics, National Research and Innovation Agency (BRIN), Komplek BRIN Jl. Sangkuriang Cisitu, Bandung 40135, Indonesia

## Abstract

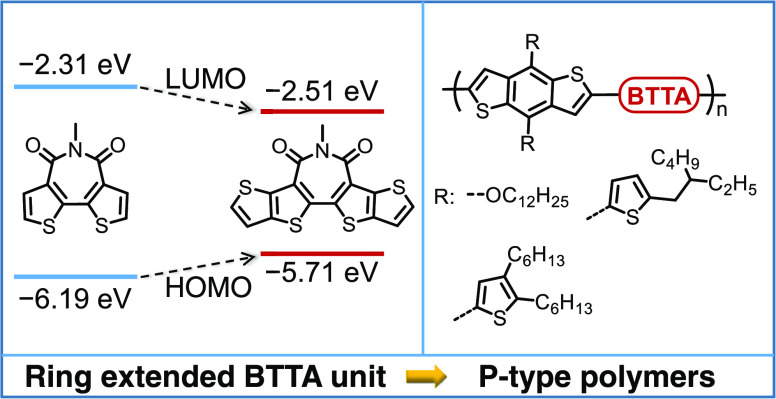

Bisthienoazepinedione (BTA) has been reported for constructing high-performing p-type conjugated polymers in organic electronics, but the ring extended version of BTA is not well explored. In this work, we report a new synthesis of a key building block to the ring expanded electron-deficient pentacyclic azepinedione (BTTA). Three copolymers of BTAA with benzodithiophene substituted by different side chains are prepared. These polymers exhibit similar energy levels and optical absorption in solution and solid state, while significant differences are revealed in their film morphologies and behavior in transistor and photovoltaic devices. The best-performing polymers in transistor devices contained alkylthienyl side chains on the BDT unit (**pBDT-BTTA-2** and **pBDT-BTTA-3**) and demonstrated maximum saturation hole mobilities of 0.027 and 0.017 cm^2^ V^–1^ s^–1^. Blends of these polymers with PC_71_BM exhibited a best photovoltaic efficiency of 6.78% for **pBDT-BTTA-3**-based devices. Changing to a low band gap non-fullerene acceptor (BTP-eC9) resulted in improved efficiency of up to 13.5%. Our results are among the best device performances for BTA and BTTA-based p-type polymers and highlight the versatile applications of this electron-deficient BTTA unit.

## Introduction

Organic semiconductors have emerged as a promising alternative to traditional inorganic electronics due to their potential for low-cost, lightweight, and flexible applications.^1−3^ Among the various organic semiconducting materials, p-type conjugated polymers with hole-transporting ability have been intensively investigated as electron donors in organic photovoltaics (OPVs) because of their tunable optoelectronic properties, high absorption coefficients, and large-scale processability.^4−6^ Generally, these polymers possess an alternating donor–acceptor (D–A) architecture, where D is an electron-rich heterocycle, and A is an electron-deficient monomer.^7^ In such a system, the choice of D and A units can significantly affect the photophysical and film-forming properties of the polymer. In OPV applications, the use of benzo[1,2-*b*:4,5-*b*′]dithiophene (BDT) as the electron-rich comonomer has become prevalent due to its combination of high solubilizing power and optimal energy levels, and the overall polymer performance has been tuned by the choice of acceptor comonomer.^8^ In the past few years, several representative electron-deficient units, such as benzo[1,2-*c*:4,5-*c*′]dithiophene-4,8-dione (BDD) and dithieno[3′,2′:3,4;2″,3″:5,6]benzo[1,2-*c*][1,2,5]thiadiazole (DTBT) have been developed to endow high-performance BDT copolymer donors PM6 and D18.^9,10^ To further enhance the performance of BDT-based polymers in single-junction and double-junction OPV cells, for both indoor and AM 1.5G applications, the development of new electron-deficient comonomers is important.^7,11−15^

**Scheme 1:**
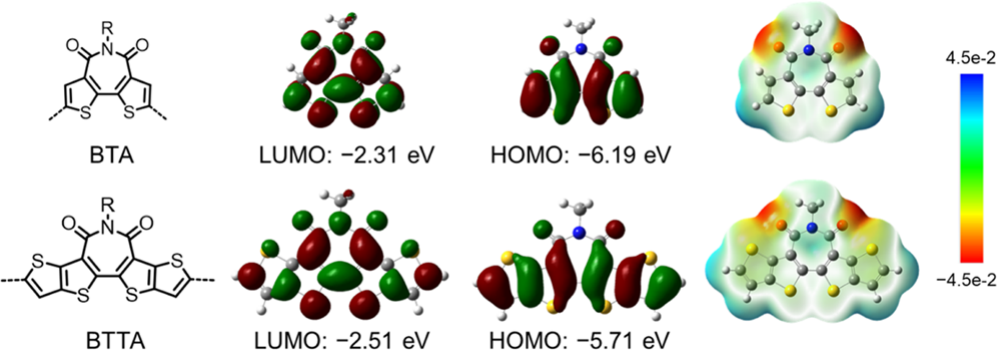
Chemical Structures, Frontier Molecular Orbital Energy Levels, and Electrostatic Potential (ESP) Surface for Bisthieno[3,2-*c*:2′,3′-*e*]azepine-4,6(5H)-dione (BTA) and Extended Pentacyclic Azepinedione (BTTA) Calculations were performed at the DFT/B3LYP/6-31G* level, and the alkyl side chains are replaced with the methyl groups for calculation simplicity.

π-Conjugated bisthieno[3,2-*c*:2′,3′-*e*]azepine-4,6(5H)-dione (BTA), as a derivative of rylene diimides and thieno[3,4-*c*]pyrrole-4,6-dione, is a promising candidate for constructing high-performance conjugated polymers in organic electronics (Scheme 1).^16^ Remarkable charge carrier mobilities have been demonstrated in transistor devices for both a BTA-homo-polymer and various BTA-based D–A copolymers, benefiting from a high degree of crystallinity and thin-film order as a result of the planarity of the BTA unit.^17^ The solar cell performance of BDT and BTA copolymers was also reported; by blending with PC_71_BM, a peak power conversion efficiency (PCE) of 5.50% was obtained in an inverted OPV device.^18^ Ring extension of the BTA unit to a pentacyclic azepinedione (bisthieno[2′,3′:4,5]thieno[2,3-*c*:2′,3′*-e*]azepine-4,6(5H)-dione, BTTA) is of particular interest, as replacing thiophene with thieno[3,2-*b*]thiophene can increase the backbone conjugation length and thus improve intermolecular π–π stacking and charge transport.^19−21^ This conjugation extension is also calculated to lead to an increase in electron affinity and a reduction in band gap (Scheme 1). Examination of the electrostatic potential (ESP) of BTA and BTTA shows a similar distribution, with negative potential distributed around the electron-withdrawing imide group and positive electrostatic potentials around the peripheral thiophenes. Although some molecular and polymeric materials containing electron-deficient BTTA units have been designed and explored as polymer acceptors in OPVs, n-type organic field-effect transistors (OFETs) or n-type organic thermoelectrics and^22−26^ their application in p-type donor polymers have been limited. Such BTTA copolymers exhibited modest PCEs of 5.46–6.18%.^27,28^ We were interested to revisit BTTA-based copolymers to explore their potential as medium gap donor polymers in OPV.

**Scheme 2:**
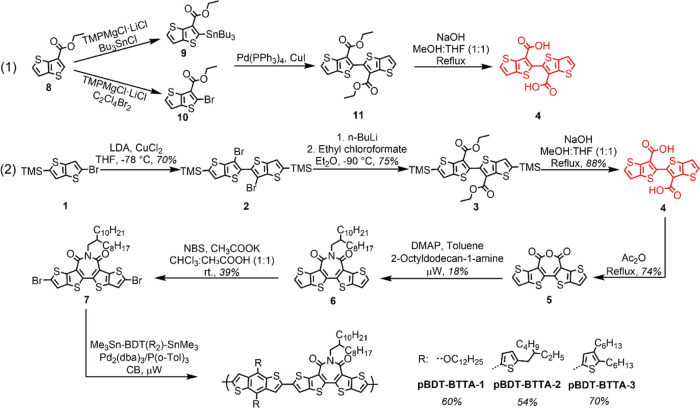
(1) Reported Route to Key Intermediate **4** and (2) Our Synthetic Route to **4** and Polymers **pBDT-BTTA-1–3**

In this work, we have designed and synthesized three BTTA-based p-type conjugated polymers, **pBDT-BTTA-1–3**, by copolymerizing distannylated BDT monomers flanked with different side chains (Scheme 2). We also report a new synthetic route to a key intermediate in the preparation of BTTA, thereby facilitating its preparation. Side-chain engineering with alkyl substitution on the BDT part was utilized for the fine-tuning of molecular properties and aggregation behavior of the D–A copolymers. With similar conjugated skeletons, these polymers show analogous optical properties and electron cloud distributions. Negligible differences between their solution and film spectra imply strong π–π stacking aggregation due to their planar backbone. All of the polymers show typical p-type charge transport behavior in OFETs, but the saturation hole mobilities of **pBDT-BTTA-2** and **pBDT-BTTA-3** are nearly 40-fold and 20-fold higher than that of **pBDT-BTTA-1**. This can be attributed to the two-dimensional conjugated structure from the introduction of alkylthienyl substituents onto the BDT backbone.^29^

To evaluate the photovoltaic performance of these polymers, we first fabricated devices with a fullerene derivative PC_71_BM as an acceptor, obtaining a high PCE of 6.78% for **pBDT-BTTA-3** after careful selection of processing solvents and additives. This value is higher than the efficiencies of **pBDT-BTTA-1–2** and the other reported BTTA-based polymers.^27,28^ Furthermore, an optimal PCE of 13.5% was reached by blending with a non-fullerene acceptor (NFA) BTP-eC9, which is the highest value for binary OSCs with a BTTA-based polymer as the donor. To match the low band gaps of current NFAs, a larger optical gap (*E*_g_^opt^ > 1.8 eV) is a prerequisite. There is still room to reach the “record efficiency” range through, for example, adjusting the alkyl substitutes and introducing thiophene bridges between BDT and BTTA units. Our results show that the electron-deficient BTTA unit is a promising candidate for developing conjugated polymers as electron donors for high-efficiency non-fullerene OPVs, and the choice of a comonomer is crucial to control film morphologies of these p-type polymers.

## Results and Discussion

### Material Synthesis and Characterization

The BTTA monomer was synthesized following modified literature procedures,^16,28^ as outlined in Scheme 2. Initial efforts were focused on the formation of [2,2′-bithieno[3,2-*b*]thiophene]-3,3′-dicarboxylic acid (**4**) as a key intermediate. In the previously reported synthetic route,^23,28^ two mono-stannylated and mono-brominated coupling partners need to be prepared from ethyl thieno[3,2-*b*]thiophene-3-carboxylate (**8**) for enabling a Pd-catalyzed Stille cross-coupling. Here, we develop an alternative route to **4**, avoiding the use of toxic tributyltin, which is based on dimerization via oxidative coupling using CuCl_2_. The whole synthetic procedures to **pBDT-BTTA-1**–**3** are outlined in Scheme 2. Thus, the treatment of the 2-bromo-5-trimethylsilylthieno[3,2-*b*]thiophene (**1**) with 1 equiv of LDA at −78 °C resulted in lithiation in the 3-position, which rapidly rearranged by the halogen dance mechanism to afford the 2-lithiated derivative. This was oxidatively dimerized *in situ* by treatment with CuCl_2_ to afford **2** in 70%. Subsequent dilithiation at −90 °C followed by a reaction with ethyl chloroformate afforded the resulting ester **3** in 75%. Attempted lithiation at higher temperatures resulted in some undesired ring-opening, and similarly, attempts to react the dilithiated material directly with carbon dioxide to afford **4** were unsuccessful, due to the reduced reactivity of CO_2_ compared to ethyl chloroformate. Conversion to diacid **4** was readily achieved by hydrolysis with sodium hydroxide. Treatment with acetic anhydride under reflux afforded the ring-closed anhydride **5** in 74%.

Due to the poor solubility of **5** in common organic solvents and the tendency of ring-opening under aqueous conditions, the crude product was used directly without purification. Reaction with branched 2-octyldodecan-1-amine in the presence of catalytic DMAP under microwave heating conditions gave **6** in 18%. The low yield is particularly attributable to use of a hindered branched amine, while similar issues were reported in BTA derivatives.^30^ Subsequent bromination with NBS gave the requisite dibrominated monomer **7** in 39%.

All polymers were synthesized via microwave-assisted^31,32^ Stille cross-coupling reactions between **7** and the corresponding distannylated BDT comonomer. The polymers were purified by precipitation in methanol and subsequent Soxhlet extraction (methanol, acetone, hexane, and chloroform) to remove the catalyst and low-molecular-weight (MW) oligomers. The chemical structures of the polymers were evaluated using ^1^H NMR and elemental analysis. Despite the use of a high boiling point, deuterated solvent (1,1,2,2-tetrachloroethane-*d*_2_, TCE-*d*_2_) at 130 °C, the ^1^H NMR spectra of the polymers were poorly resolved and difficult to assign, especially for **pBDT-BTTA-1** in which no aromatic signals were observed. This can be related to the low concentration of the polymer in solution (*ca*. 4.5 mg mL^–1^), as well as strong intermolecular aggregation.

The solubility of the polymers in chlorobenzene (CB) at room temperature was also low, particularly for **pBDT-BTTA-1**. As such, the MW of **pBDT-BTTA-1** was determined by gel permeation chromatography (GPC) in 1,2,4-trichlorobenzene (TCB) at 140 °C. For **pBDT-BTTA-2** and **pBDT-BTTA-3**, the solubility was sufficient to enable MWs to be measured in CB at 80 °C. As shown in Table 1, the number-average MW (*M_n_*) of **pBDT-BTTA-1** was considerably lower than the other polymers. This is most likely a result of poor solubility, resulting in early precipitation during the polymerization. The thermal stability of the polymers was probed by thermogravimetric analysis (TGA) (see the Supporting Information and Table 1), in which a 5% weight loss was observed at 313 °C for **pBDT-BTTA-1**, and at 419 °C for **pBDT-BTTA-2** and **pBDT-BTTA-3**. The lower stability of **pBDT-BTTA-1** could be related to the alkoxy side chains on the BDT ring. The thermal properties of the polymers were evaluated using differential scanning calorimetry (DSC) measurements. As shown in Figure S5, no obvious thermal transitions were observed in the second heating and cooling scans of the polymers recorded between −30 and 330 °C under nitrogen.

**Table 1:** Molecular Weights, Thermal and Optical Properties, and Electronic Energy Levels of **pBDT-BTTA-1**–**3**

polymer	*M_n_*^a^ [kDa]	*M*_w_^a^ [kDa]	*Đ*^a^	*T*_d_^c^ [°C]	λ_max_^sol^^d^ [nm]	λ_max_^film^^e^ [nm]	*E*_g_^opt^^f^ [eV]	I.P.^g^ [eV]	LUMO^h^ [eV]
**pBDT-BTTA-1**	9^b^	26^b^	2.8^b^	313	580 (632)	580 (629)	1.74	5.30	–3.56
**pBDT-BTTA-2**	50	163	3.3	419	638 (589)	642 (590)	1.75	5.20	–3.45
**pBDT-BTTA-3**	42	172	4.1	419	630 (584)	638 (593)	1.75	5.26	–3.51

a Determined by GPC in CB at 80 °C against polystyrene standards with the exception of

b which was determined in TCB at 140 °C.

c Decomposition temperature measured by TGA.

d Measured in dilute CB (10^–5^ M) at 20 °C. Shoulder peaks in parentheses.

e Thin films spin-coated on glass substrates from 5 mg mL^–1^ CB solution. Shoulder peaks in parentheses.

f Optical band gap estimated from the onset of film absorption.

g Determined as a thin film by UV-PESA (error ± 0.05 eV).

h Estimated with equation: *E*_g_^opt^ = I.P. + LUMO.

### Optoelectrical Properties and Theoretical Calculations

**Figure 1:**
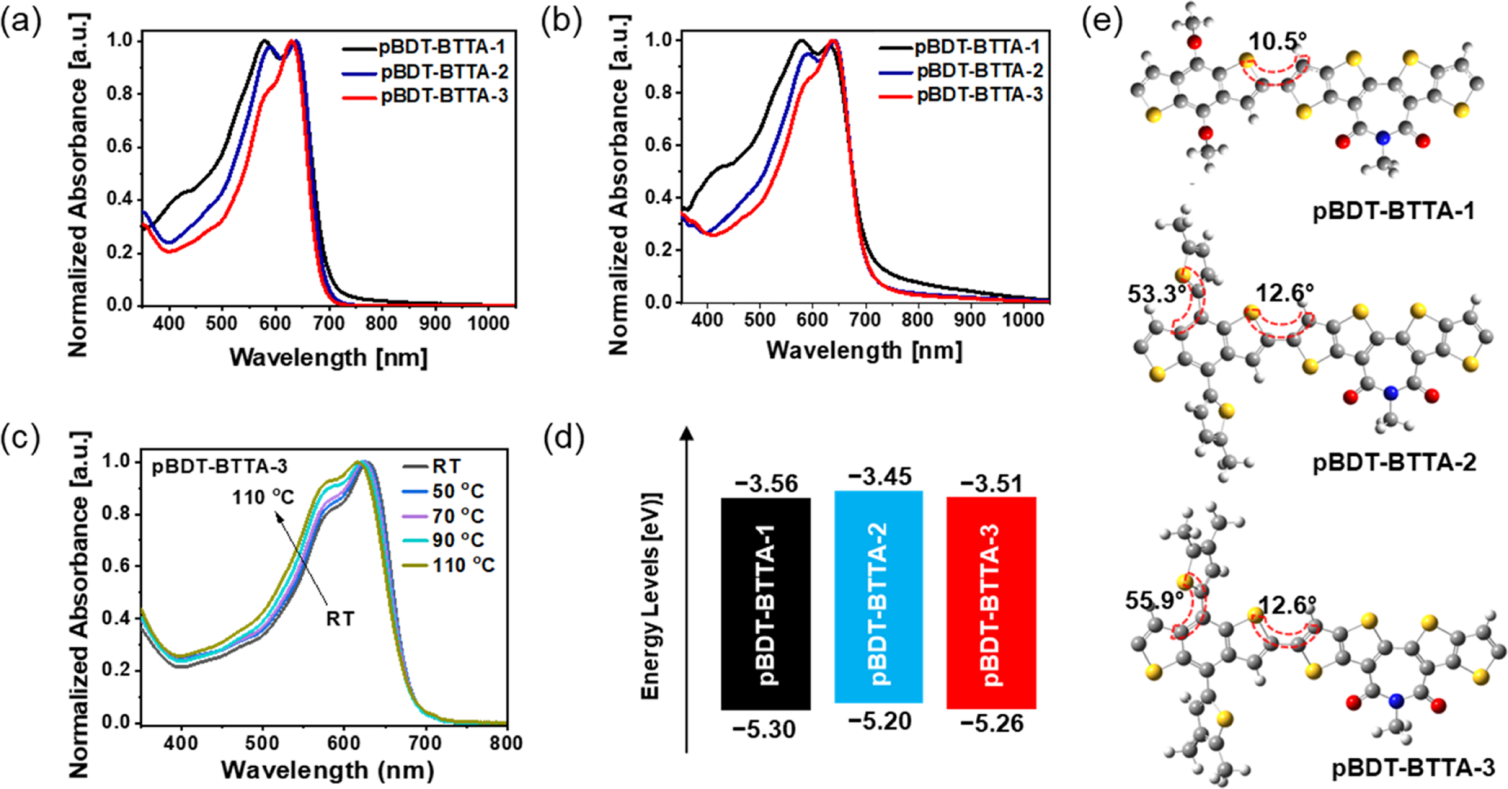
Normalized UV–vis absorption spectra in (a) dilute CB solution (10^–5^ M) at 20 °C and (b) spin-coated thin films from CB solution (5 mg mL^–1^) of **pBDT-BTTA-1**–**3**. (c) Normalized temperature-dependent absorption spectra of **pBDT-BTTA-3**. (d) Schematic diagram of the electronic energy levels and (e) optimized geometries (one repeating unit) of **pBDT-BTTA-1–3**.

The optical properties of the polymers in dilute CB and as spin-coated thin films were investigated by ultraviolet–visible (UV–vis) absorption spectroscopy, as presented in Figure 1 and Table 1. The solutions of all three polymers are broadly similar (Figure 1a), with peak onsets around 700 nm and two major absorption peaks present (around 580 and 635 nm). There are subtle differences in the relative intensity of these two peaks for the three polymers. **pBDT-BTTA-1** shows a higher energy peak λ_max_^sol^ at 580 nm, with a lower energy shoulder at 632 nm, while the opposite is observed for **pBDT-BTTA-2** and **pBDT-BTTA-3**, with the most intense peaks located at 638 and 630 nm, with their shoulder peaks at 589 and 584 nm, respectively. The longer wavelength peak is often associated with an extended polymer backbone and aggregation in solution, which can be influenced by the inclusion of the alkylthienyl side chains on the BDT portion of the polymers. This increase in intermolecular π–π interactions is consistent with previous reports, comparing alkoxy- and alkylthienyl-substituted BDT polymers.^33^ The relative intensity of the longer wavelength peak is larger for **pBDT-BTTA-3** than **pBDT-BTTA-2**, which is ascribed to the linear nature of the side chains in the former versus the branched side chains of the latter.

All of the thin-film spectra closely resemble the solution (Figure 1b), suggesting that the polymers are already aggregated extensively in solution. The “pre-aggregation” behavior in the solution state could be beneficial for realizing nanoscale phase separation when blending with NFAs.^34^ Temperature-dependent absorption characterization was also utilized to verify the strong intermolecular interactions of the polymers (Figure 1c). With increasing solution temperature, the absorption of **pBDT-BTTA-3** gradually blue-shifted, and the ratio of the two peaks changed.

The ionization potentials of thin films of **pBDT-BTTA-1**, **pBDT-BTTA-2**, and **pBDT-BTTA-3** were measured using photoelectron spectroscopy in air (PESA) to be 5.30, 5.20, and 5.26 eV, respectively (Figure 1d and Table 1). It seems that the replacement of the alkoxy group with electron-rich alkylthienyl side chains in **pBDT-BTTA-2** and **pBDT-BTTA-3** results in a small decrease in ionization potential, suggesting that the alkylated thienyl groups are overall more electron-donating than the alkoxy group. There is a slight difference of 0.06 eV between the ionization potential of **pBDT-BTTA-2** and **pBDT-BTTA-3**, which may be the result of differences in steric hindrance, with the addition of the dialkylthienyl side chains in **pBDT-BTTA-3** resulting in a more torsionally twisted structure than **pBDT-BTTA-2**. Here, we note that the values are close, considering the error of the technique (±0.05 eV). Theoretical calculation found that the energy levels of **pBDT-BTTA-2** and **pBDT-BTTA-3** are upshifted compared to those of **pBDT-BTTA-1** (Figure S6–8), in agreement with the PESA results. All polymers are predicted to exhibit little torsional twisting between the BDT and the adjacent BTTA units (Figure 1e).

### OFET Devices

**Figure 2:**
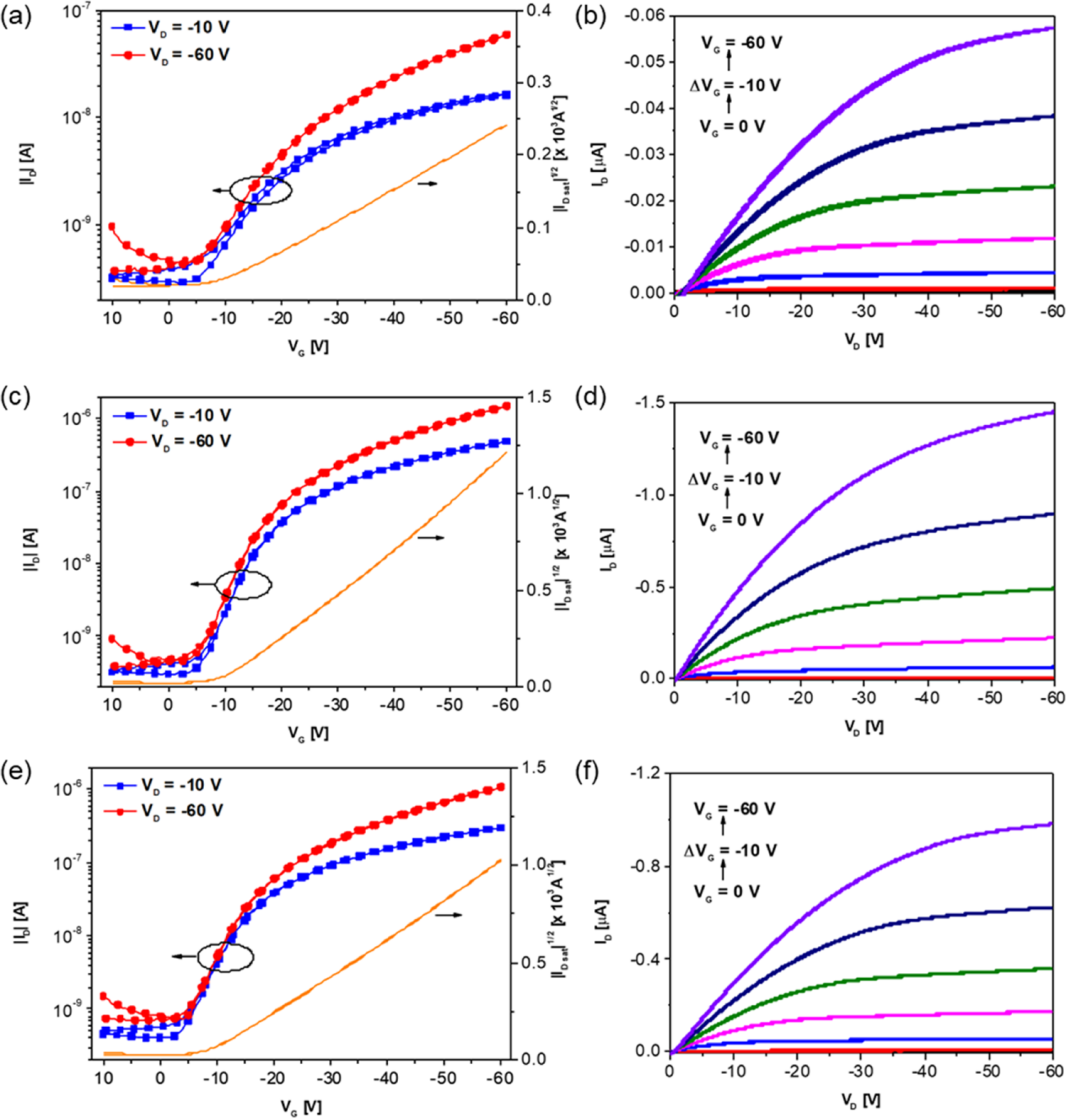
Transfer (left) and output (right) characteristics of the best performing TG-BC devices of **pBDT-BTTA-1** (a, b), **pBDT-BTTA-2** (c, d), and **pBDT-BTTA-3** (e, f), fabricated using Au (40 nm) as source and drain electrodes, treated with PFBT and a CYTOP dielectric. Polymer active layers were spin-coated from CB solution (5 mg mL^–1^) and annealed at 120 °C for 30 min. The channel width and length were 1000 and 40 μm, respectively.

The charge transport properties of the polymers were evaluated in top-gate bottom-contact (TG-BC) field-effect transistors. Devices were fabricated using gold source–drain electrodes and a cyclic transparent optical polymer (CYTOP) dielectric. To lower the work function of the gold and aid hole injection, the electrodes were treated with a self-assembled monolayer (SAM) of pentafluorobenzenethiol (PFBT) prior to polymer deposition. Figure 2 shows the transfer and output characteristics of the best-performing devices. In all cases, unipolar, p-type charge transport was observed, with negligible hysteresis between the forward and reverse gate voltage sweeps. As presented in Table 2, **pBDT-BTTA-1** exhibited the lowest device performance, with an average saturated charge carrier mobility (μ_sat_) of 6.8 × 10^–4^ cm^2^ V^–1^ s^–1^. This can be partially attributed to the difficulties in forming a homogeneous film during device fabrication, due to the low molecular weight and poor solubility. The presence of large aggregates becomes detrimental to charge transport (atomic force microscopy (AFM) topography images of **pBDT-BTTA-1**, *vide infra*), and the low MW of the polymer can limit the charge transport.^35^ In contrast, the μ_sat_ values of **pBDT-BTTA-2** and **pBDT-BTTA-3** were considerably increased to 0.023 and 0.013 cm^2^ V^–1^ s^–1^, respectively. This is likely due to a combination of the improved solubility and film-forming ability of these two polymers and the 2D-conjugated structures afforded by the alkylthienyl side chains, which provide enlarged π-overlap between the adjacent backbones.^36,37^

**Table 2:** Device Performance of OFETs-Based **pBDT-BTTA-1**–**3**^f^^a^

polymer	μ_sat_ [×10^–2^ cm^2^ V^–1^ s^–1^]	*V*_T_ [V]	*I*_on_/*I*_off_
**pBDT-BTTA-1**	0.068 ± 0.0088 (0.076)	–6.4 ± 5.8	10^2^–10^3^
**pBDT-BTTA-2**	2.3 ± 0.26 (2.7)	–12.7 ± 0.8	10^3^–10^4^
**pBDT-BTTA-3**	1.3 ± 0.27 (1.7)	–12.8 ± 0.5	10^3^–10^4^

a TG-BC devices fabricated using Au (40 nm) source–drain electrodes treated with PFBT and a CYTOP dielectric. Polymer thin layers were spin-coated from CB solution (5 mg mL^–1^) and annealed at 120 °C for 30 min. The maximum saturation mobility values are in parentheses.

### OPV Devices

**Figure 3:**
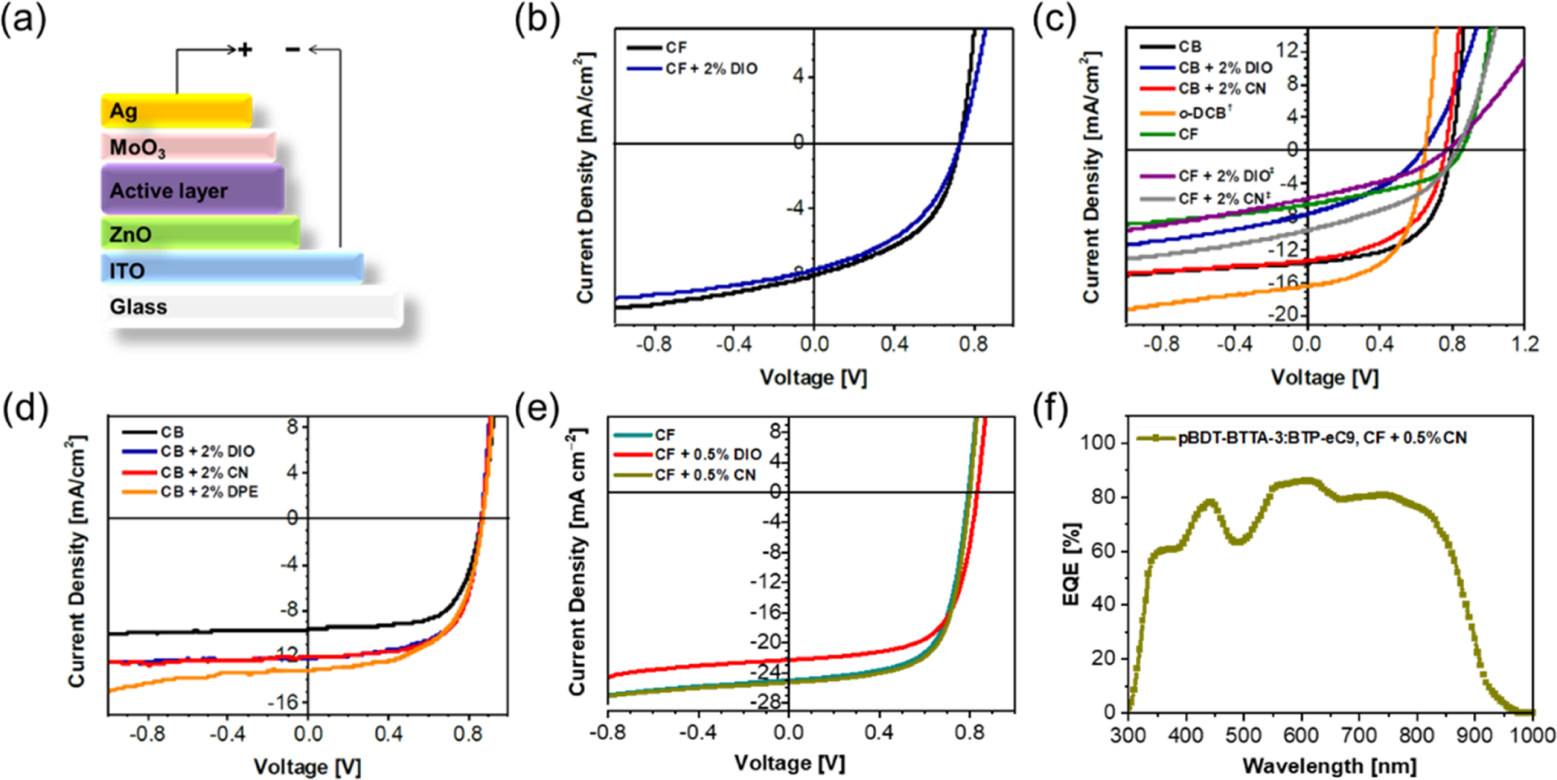
(a) Schematic diagram of inverted device structure (ITO/ZnO/**pBDT-BTTA-1–3**:PC_71_BM or BTP-eC9/MoO_3_/Ag). *J–V* curves of (b) **pBDT-BTTA-1**:PC_71_BM, (c) **pBDT-BTTA-2**:PC_71_BM, (d) **pBDT-BTTA-3**:PC_71_BM and (e) **pBDT-BTTA-3**: BTP-eC9, measured under AM 1.5G illumination at 100 mW cm^–2^. The pixel size was 0.045 cm^2^. (f) EQE curve of the optimal OPV device based on **pBDT-BTTA-3**: BTP-eC9.

The photovoltaic performances of the polymers were investigated as donors in blends with both a fullerene and a non-fullerene acceptor in an inverted configuration (ITO/ZnO/**pBDT-BTTA-1**–**3**:PC_71_BM or BTP-eC9/MoO_3_/Ag) (Figure 3a). Figure 3b–e displays the *J–V* characteristics of these devices, and the data are summarized in Table 3 and Tables S1 and S2. For initial testing with fullerene acceptor, the photoactive layers, consisting of **pBDT-BTTA-1**–**3**:PC_71_BM in a blend ratio (w:w) of 1:2, were spin-coated at 3000 rpm from their respective CB solutions (24 mg mL^–1^) without any solvent additives. We note that due to the poor solubility of **pBDT-BTTA-1** in CB, devices were prepared from chloroform. Poor performance with PCE up to 2.75% was found, which we mainly attribute to the low molecular weight and difficulties in forming homogeneous films. In contrast, **pBDT-BTTA-2**:PC_71_BM and **pBDT-BTTA-3**:PC_71_BM blends exhibited promising performance under the same initial screening, with better PCEs of 5.58 and 5.17%, respectively. Subsequently, 1,8-diiodooctane (DIO) or 1-chloronaphthalene (CN) were utilized as high boiling point solvent additives in an attempt to improve device performance.^38^ The efficiencies of **pBDT-BTTA-2**-based devices dropped with either DIO or CN. Further efforts to improve its OPV performance were made through adjusting processing solvents, spin-coating speeds, and additives. However, in all cases, the OPV efficiencies decreased, particularly for devices spin-coated from chloroform solutions (1.32–2.34%). This can be related to different Hansen solubility parameters of the processing solvents, which can affect the blend morphologies during film formation.^39^ On the contrary, for **pBDT-BTTA-3**, adding DIO or CN additives could well help to tune the blend film morphology and enable a higher *J*_SC_ (11.30–13.20 mA cm^–2^). The efficiencies of **pBDT-BTTA-3**:PC_71_BM devices were successfully increased to 6.78%, which is the best polymer/fullerene system among the three polymers and also a record value for BTTA-based p-type polymers in OPV devices to the best of our knowledge.

**Table 3:** Optimized Photovoltaic Parameters of **pBDT-BTTA-1**–**3**-Based Devices

active layer	*J*_SC_ [mA cm^–2^]	*V*_OC_ [V]	FF	PCE [%]^a^	*E*_loss_ [eV]^b^
**pBDT-BTTA-1**:PC_71_BM	7.88 ± 0.13 (7.99)	0.67 ± 0.13 (0.73)	0.44 ± 0.08 (0.47)	2.37 ± 0.76 (2.75)	1.07
**pBDT-BTTA-2**:PC_71_BM	12.90 ± 0.50 (13.58)	0.78 ± 0.03 (0.79)	0.55 ± 0.04 (0.56)	5.58 ± 0.64 (6.09)	0.97
**pBDT-BTTA-3**:PC_71_BM	11.40 ± 0.50 (12.10)	0.86 ± 0.06 (0.86)	0.63 ± 0.02 (0.65)	6.27 ± 0.38 (6.78)	0.89
**pBDT-BTTA-3**:BTP-eC9	24.20 ± 0.50 (24.40)	0.82 ± 0.10 (0.82)	0.65 ± 0.02 (0.65)	13.2 ± 0.30 (13.5)	0.51

a Average values and standard deviations were obtained from 10 devices, which were expressed as mean ± SD. Optimal results are listed in parentheses.

b *E*_loss_ was estimated by *E*_g_ – e*V*_OC_, where *E*_g_ is the smallest value between the donor and acceptor.

Based on the promising performance of **pBDT-BTTA-3** with investigated devices using a low band gap non-fullerene acceptor BTP-eC9,^40^ different additives and annealing temperatures were explored to optimize the blend film morphology (Table S3). The best efficiency of 13.5% was obtained for **pBDT-BTTA-3**:BTP-eC9 devices with 0.5% CN but without thermal treatments. A higher short-circuit current density (*J*_SC_) of 24.2 mA cm^–2^ was achieved due to the absorption contribution from the non-fullerene acceptor BTP-eC9, which can be clearly observed in the external quantum efficiency (EQE) of the optimal device (Figure 3f). The calculated *J*_SC_ from the EQE is 23.62 mA cm^–2^, which agrees well with the corresponding *J*_SC_ from *J–V* curves. Compared to the fullerene-based devices, a much lower energy loss of 0.51 eV was also achieved in these non-fullerene OPV devices (Table 3).

Correlating with the optimized performance of **pBDT-BTTA-3**:BTP-eC9 devices, Figure S9 and Table S3 provide the hole mobilities of the neat **pBDT-BTTA-3** and the hole and electron mobility of **pBDT-BTTA-3**:BTP-eC9 blend determined by space-charge-limited current (SCLC) approach (see experimental details in the Supporting Information). The obtained hole mobilities for neat **pBDT-BTTA-3** were around 5 × 10^–4^ cm^2^ V^–1^ s^–1^, which is slightly reduced upon mixing with BTP-eC9 to 2∼4 × 10^–4^ cm^2^. This is around 2 times lower than the electron mobility of the blend, which could explain the relatively suboptimal FF values (0.65) of the **pBDT-BTTA-3**:BTP-eC9 devices.

### Morphology Analysis

**Figure 4:**
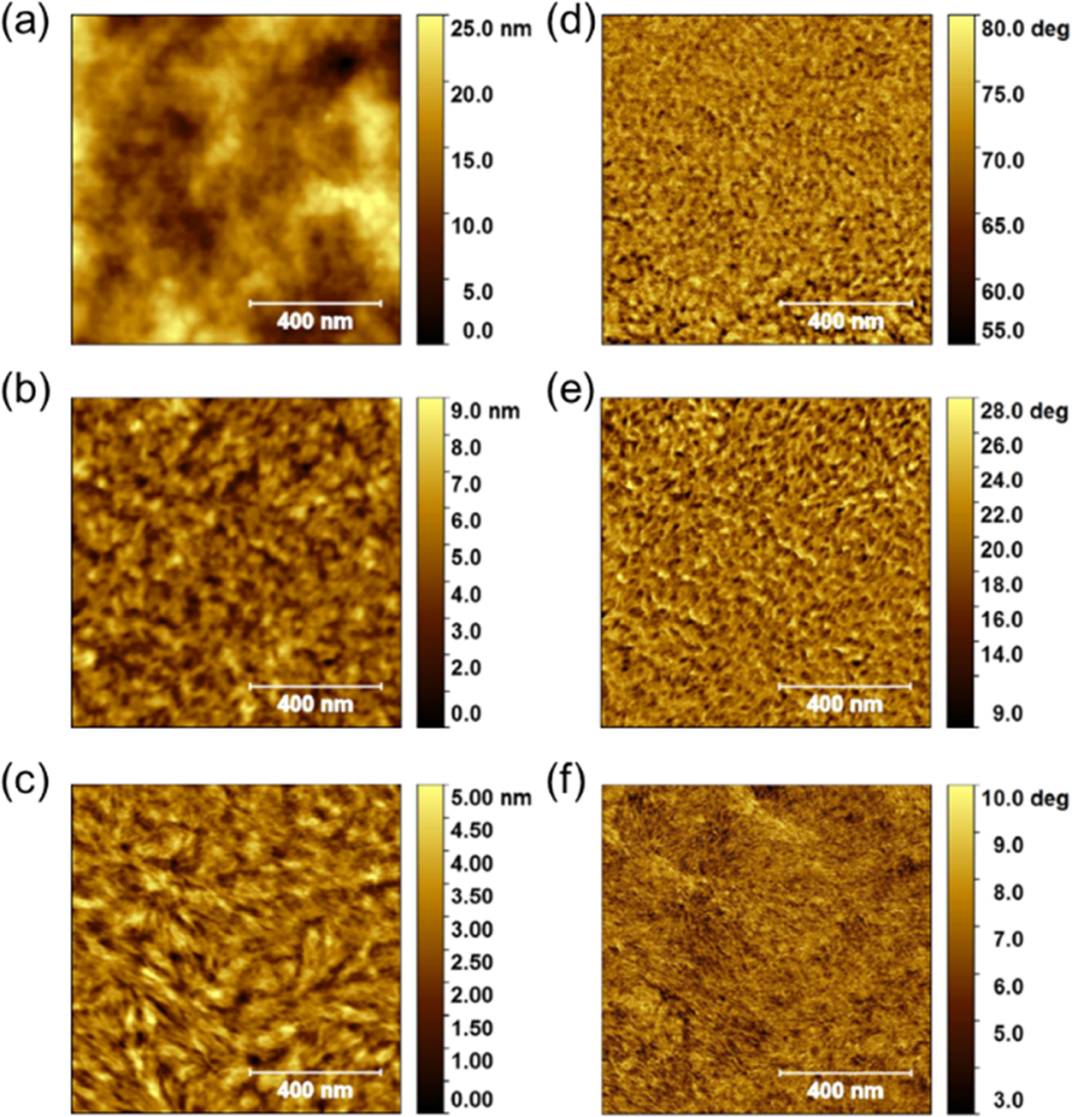
AFM topography (a–c) and phase (d–f) images of neat thin films of **pBDT-BTTA-1** (a, d), **pBDT-BTTA-2** (b, e), and **pBDT-BTTA-3** (c, f), spin-coated from CB solution (5 mg mL^–1^) and annealed at 120 °C for 30 min. Scale: 1 μm × 1 μm.

The morphologies of the neat polymers were studied by using atomic force microscopy (AFM) in tapping mode, prepared under the same conditions as those used for OFET device fabrication. As illustrated in Figure 4, the inherent differences between the side chains on the BDT monomer had a significant influence on the film structures, with both **pBDT-BTTA-2** and **pBDT-BTTA-3** exhibiting a more homogeneous morphology than **pBDT-BTTA-1**. In addition, **pBDT-BTTA-2** and **pBDT-BTTA-3** have smoother and more continuous morphologies with the root-mean-square (RMS) surface roughnesses of 1.13 nm and 0.79 nm, lower than that of alkoxy-substituted **pBDT-BTTA-1** (RMS = 4.03 nm). The substantial increase in surface roughness observed for **pBDT-BTTA-1** is probably caused by a combination of difficulties in filtering the polymer during film preparation and early precipitation during the film-forming process, which both relate to the intrinsic poor solubility of this polymer. The different morphologies of the neat films of the three polymers are in agreement with previous studies comparing alkoxy and alkylthienyl side chains on BDT containing polymers, with the alkylthienyl groups found to promote intermolecular ordering.^36,42^

**Figure 5:**
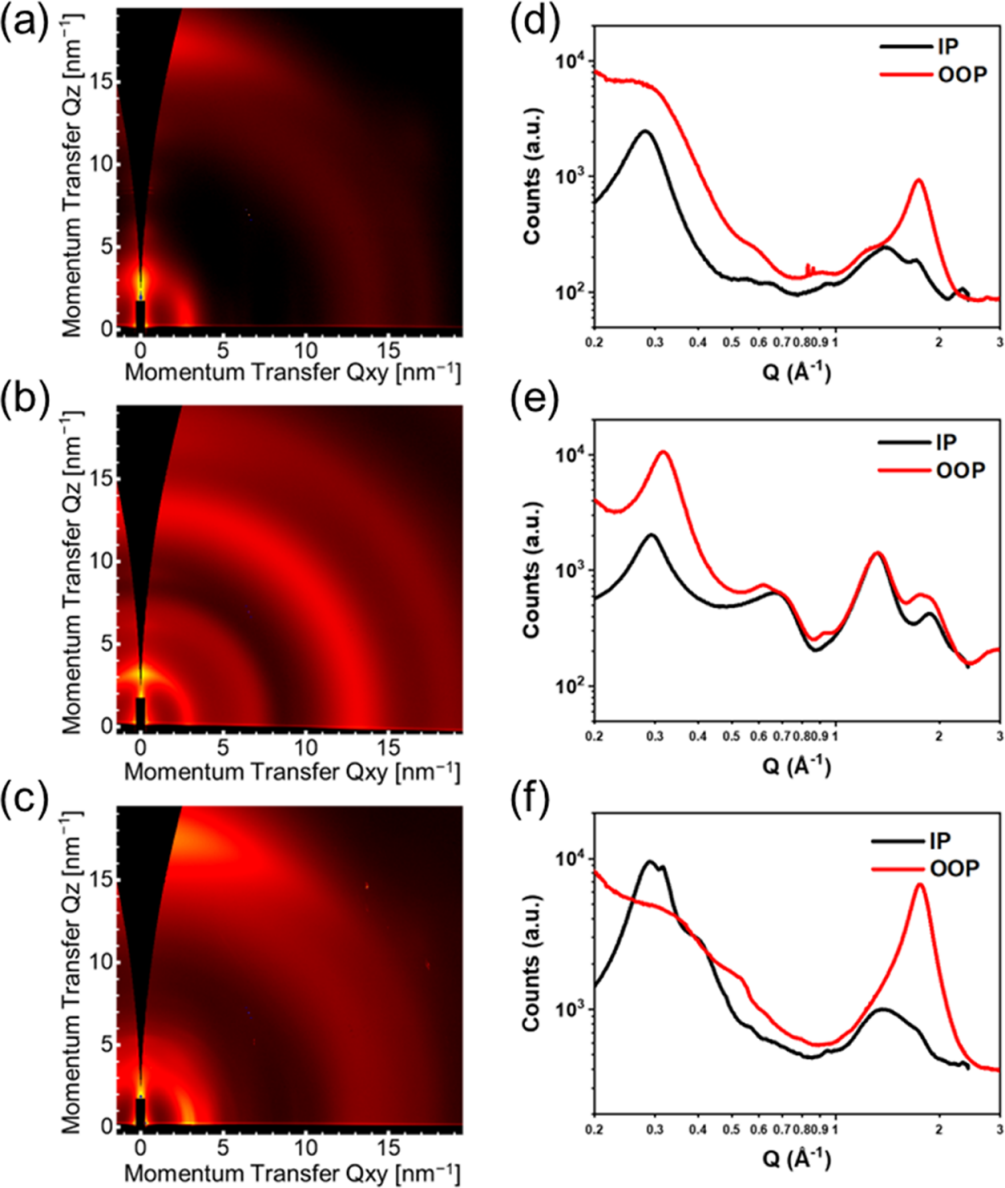
2D GIWAXS patterns (a–c) and scattering profiles (d–f) of in-plane and out-of-plane for **pBDT-BTTA-3** (a, d), **pBDT-BTTA-3**:PC_71_BM (1:2, w/w) (b, e), and **pBDT-BTTA-3**:BTP-eC9 (1:1.2, w/w) (c, f) as-spun.

GIWAXS was also performed on neat **pBDT-BTTA-3** (Figure 5a,d) and in blends with PC_71_BM (Figure 5b,e) and BTP-eC9 (Figure 5c,f). Neat **pBDT-BTTA-3** has a semicrystalline microstructure consistent with the fibrillar morphology observed with AFM. **pBDT-BTAA-3** exhibits a preferential face-on microstructure, characterized by a prominent in-plane lamellar stacking peak at *q*_*xy*_ = 0.28 Å^–1^ and out-of-plane π–π stacking peak at *q*_*z*_ = 1.74 Å^–1^, corresponding to *d*-spacings of 22.4 and 3.6 Å for lamellar stacking and π–π stacking, respectively. In the blend with PC_71_BM, **pBDT-BTAA-3** crystallites instead adopt a predominant edge-on orientation, evidenced by the lamellar stacking peak being much stronger in the out-of-plane direction. Broad rings corresponding to scattering from aggregated PC_71_BM can also be seen. In the blend with BTP-eC9, **pBDT-BTTA-3** crystallites retain their preferential face-on orientation that is seen in neat films. Additional scattering features are also seen (for example, a peak at *q*_*xy*_ = 0.32 Å^–1^) that can be assigned to BTP-eC9, indicating that the non-fullerene acceptor also shows semicrystalline character in the blend with **pBDT-BTTA-3**. The **pBDT-BTTA-3**:BTP-eC9 blend also exhibits an enhanced out-of-plane π–π stacking peak that is shifted to slightly higher q, which also provides evidence for face-on oriented BTP-eC9 crystallites in the blend. Compared to the **pBDT-BTTA-3**:PC_71_BM blend, the face-on orientation of both donor and acceptor components in the **pBDT-BTTA-3**:BTP-eC9 blend could help explain its higher photovoltaic performance.

## Conclusions

A series of new BTTA-based p-type polymers were designed and synthesized utilizing a new synthetic route to the BTTA monomer. The choice of BDT comonomer was shown to strongly influence the solubility and film-forming ability of the conjugated polymers, with more subtle effects upon the optical and electronic properties. The alkoxy-substituted BDT copolymer (**pBDT-BTTA-1**) exhibited poor solubility, resulting in inhomogeneous thin films with increased surface roughness compared to the alkylthienyl-substituted derivatives (**pBDT-BTTA-2–3**). Significant differences were observed in their performance in organic transistor devices, with the alkylthienyl polymers exhibiting greater than twentyfold improvement in charge carrier mobility compared to the alkoxy polymer, likely as a result of their better film-forming ability and the presence of conjugated alkylthienyl side chains providing additional intermolecular π–π interactions. The photovoltaic performances of these p-type conjugated polymers were also investigated as electron donors, blended with both fullerene and non-fullerene acceptors. **pBDT-BTTA-3**:PC_71_BM and **pBDT-BTTA-3**:BTP-eC9 blend films showed optimal PCEs of 6.78% and 13.5%, respectively, among the best values for BTTA-based p-type polymers so far. These results further demonstrate the potential of BTTA as an electron-deficient unit for constructing p-type conjugated polymers.

## Supporting Information Available

Synthesis of the monomers (**2**–**7**) and polymers (**pBDT-BTTA-1**–**3**); theoretical calculations; measurements details; device fabrication; TGA, DSC, and SCLC; and full photovoltaic data (PDF)

## Notes

Parts of this work have been published in a thesis (doi.org/10.25560/73009).
