# Supplementary material for: p-Type Conjugated Polymers Containing Electron-Deficient Pentacyclic Azepinedione
Source: Macromolecules. 2023 Jul 26;56(15):5825–34. doi: 10.1021/acs.macromol.3c00843 (PMC10413964; doi:10.1021/acs.macromol.3c00843)
Supplement: Supplementary file 1 — ma3c00843_si_001.pdf [file ma3c00843_si_001.pdf]

Supporting Information

p-Type Conjugated Polymers Containing Electron-Deficient Pentacyclic Azepinedione

Qiao He, Jessica Shaw, Yuliar Firdaus, Xiantao Hu, Bowen Ding, Adam V. Marsh, Alexandre S. Dumon, Yang Han, Zhuping Fei, Thomas D. Anthopoulos, Christopher R. McNeill and Martin Heeney*

All starting materials and reagents were purchased from commercial sources (Derthon OPV Co Ltd, Sigma Aldrich, TCI) and used as received, unless otherwise stated. All reactions were conducted under argon using standard Schlenkline techniques and anhydrous solvents, as commercially supplied. All microwave experiments were performed in a Biotage Initiator v2.3. Flash column chromatography was performed on silica gel (Merck Kieselgel 60 grade 40-63 μm F254). Thin layer chromatography was performed on Merck Kieselgel 60 F254 aluminum sheets and observed under 254 or 365 nm UV light.

The ^{1}H and ^{13}C NMR spectra for chemical intermediates were recorded on a Bruker AV-400 (400 MHz) spectrometer at 20 °C in chloroform-d. The ^{1}H NMR spectra for polymers were recorded on a Bruker AV-400 (400 MHz) spectrometer at 130 ° C in 1,1,2,2-tetrachloroethane-d2 (TCE-d2). Chemical shifts are reported in ppm relative to residual protons in the deuterated solvent used and coupling constants are reported in Hz.

Number-average and weight-average molecular weights, and polydispersities were determined with either an Agilent Technologies 1260 Infinity Analytical GPC, using two PL mixed B columns in series, in chlorobenzene at 80°C. The machines were calibrated against narrow weight polydispersity polystyrene standards. In cases where polymer solubility was an issue, a Polymer Laboratories PL-GPC-220 machine was used in trichlorobenzene (TCB) at 140 °C.

Electron ionization mass spectrometry was performed with a Micromass AutoSpec Premier Instrument. Electrospray ionization mass spectrometry was performed with a Micromass LCT Premier Instrument. MALDI-ToF mass spectrometry was performed with a Micromass MALDI micro MX instrument. Elemental analysis was carried out using a Thermo Flash 2000 machine.

Solution and solid-state UV-vis absorption spectra were recorded on a UV-1800 Shimadzu UV-vis spectrometer. Polymer thin films were spin-coated on glass substrates from chlorobenzene (5mg mL^{-1}) using a Laurell WS-400BZ-6NPP/LITE spin coater. Photoelectron spectroscopy in air measurements were recorded with a Riken Keiki AC-2 PESA spectrometer with a power setting of 5 nW and a power number of 0.5. Thermal gravimetric analysis plots were obtained with a PerkinElmer Pyris 1 TGA machine at a scan rate of 10 °C min^{-1}, under a nitrogen atmosphere. Differential scanning calorimetry experiments were carried out with a TA Instruments DSC TZero Q20 v24.10 instrument at a scan rate of 10 °C min^{-1} and analysed using TA Instruments Universal Analysis 2000 v4.5A software. Density functional theory calculations were carried out using the Gaussian 09 program and a basis set of 6-311G(D).[1, 2]

Atomic force microscopy images were obtained with a Picoscan PicoSPM LE scanning probe in tapping mode, under ambient conditions. Polymer thin films were prepared according to the method used when fabricating the respective transistors.

Grazing-Incidence Wide-Angle X-ray Scattering (GIWAXS).
GIWAXS measurements were performed at the SAXS/WAXS beamline at the Australian Synchrotron.[3] Two-dimensional scattering patterns were recorded on a Dectris Pilatus 1M detector with 11 keV photons used to probe the samples. The total exposure time was 3 s, with the reported images a composite of three separate 1 s exposures taken with different lateral detector offsets to fill in the regions missed by gaps in the detector. A silver behenate standard was used to calibrate the sample-to-detector distance. Results were analysed using NIKA 2D implemented in IgorPro.

Transistor Device Fabrication and Characterization
All film preparation was carried out under inert atmosphere. TG-BC devices were fabricated on glass substrates using Au (40 nm) source-drain electrodes and CYTOP dielectric. Au electrodes were treated with PFBT to form a self-assembled monolayer to increase the work function. Polymers were dissolved in chlorobenzene at a concentration of 5mg mL^{-1} (**pBDT-BTTA-2** and **pBDT-BTTA-3** were filtered, whereas **pBDT-BTTA-1** could not pass through the 0.45 μm PTFE filter), and spin-coated at 2000 rpm for 60 s, from a hot solution. The obtained polymer films were annealed at 120 °C for 30 min before the dielectric was spin-coated. The channel width and length of the transistors was 1000 μm and 40 μm, respectively. Transistor characterization was carried out under nitrogen using a Keithley 4200 parameter analyser. Mobility was extracted from the slope of $I_D^{1/2}$ vs. V_G.

OPV Device Fabrication and Characterization
BHJ solar cells were fabricated with inverted (ITO/ZnO/Polymer:$PC_{71}BM$ or BTP-eC9/MoO_3/Ag) configuration and tested under simulated 100 mW cm^{-2} AM 1.5G illumination using a Xenon lamp (Oriel Instruments). Prior to deposition, the ITO substrates were cleaned (detergent, acetone and IPA) and subjected to an oxygen plasma treatment at 100 W for 7 min. The ZnO layer was prepared using the sol-gel method[4, 5] from a solution of zinc acetate dehydrate (219.5 mg), 2-methoxyethanol (2 mL) and ethanolamine (0.06 mL), spin-coated onto glass and annealed at 150 °C for 20 min. In all cases polymer active layers were prepared with a donor:acceptor (polymer:$PC_{71}BM$) blend ratio of 1:2 (w:w) (24 mg mL^{-1}) and spin-coated at either 1000, 2000, or 3000 rpm from chloroform, chlorobenzene, or *o*-dichlorobenzene. The MoO_3 (10 nm) and Ag (100 nm) layers were then deposited by thermal evaporation through a shadow mask. The pixel size, defined by the spatial overlap of the anode and cathode was 0.045 cm^2.

Space-Charge-Limited Current (SCLC) Device Fabrication and Characterization
Hole-only diode configuration: Glass/ITO/PEDOT:PSS/**pBDT-BTTA-3** or **pBDT-BTTA-3**:BTP-eC9/MoO_3/Ag. *Electron-only diode configuration*: Glass/ITO/ZnO/**pBDT-BTTA-3**:BTP-eC9/PFN-Br/Ag. The active layers were prepared following the optimized condition of OPV devices described above with different spin speeds to obtain different active layer thicknesses. Active-layer thicknesses were measured with a Tencorsurface profilometer. The other layers were prepared following the established procedures.[6] The SCLC mobility was estimated was estimated using Equation E1:[7]

$$J(V) = \frac{9}{8}\varepsilon_0\varepsilon_r\mu_0 \exp\left(0.89\beta\sqrt{\frac{V-V_{bi}}{L}}\right)\frac{(V-V_{bi})^2}{L^3} \quad \text{(E1)}$$

Definition	Variable	Units
zero-field mobility	μ_0	$cm^2\ V^{-1}\ s^{-1}$
film thickness	L	cm
dark current density	J	$mA\ cm^{-2}$
voltage	V	V
built in voltage	V_{bi}	V
vacuum permittivity	ε_0 (88.54×10^{-12})	$mA\ s\ V^{-1}\ cm^{-1}$
dielectric constant	ε_r (3)	
field activation factor	β	$cm^{1/2}\ V^{-1/2}$

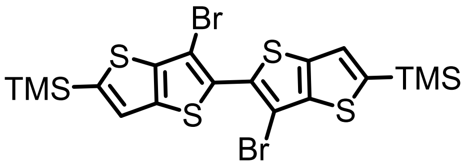

(3,3'-dibromo-[2,2'-bithieno[3,2-b]thiophene]-5,5'-diyl)bis(trimethylsilane) (**2**)

Lithium diisopropylamide (1.2 M solution in THF, 36.8 mmol) was added dropwise to a stirred solution of (5-bromothieno[3,2-b]thiophen-2-yl)(trimethyl)silane[8] (10.0 g, 34.3 mmol) in anhydrous tetrahydrofuran (150 mL) at −78 °C, under an argon atmosphere. After 30 min, copper(II) chloride (4.9 g, 36.8 mmol) was added and the reaction mixture was allowed to warm to room temperature over 16 h. Any remaining copper(II) chloride was then removed by vacuum filtration and the resulting solution was extracted with diethyl ether (50 mL). The combined organics were combined, washed with water (3 × 50 mL), brine (50 mL), dried ($MgSO_4$) and the volatiles removed *in vacuo*. Purification of the crude product by column chromatography over silica (eluent:hexane) yielded a yellow solid (5.3 g, 9.1 mmol, 53%). ^1H NMR (400 MHz, $CDCl_3$) δ: 7.40 (2H, s, ArH), 0.38 (18H, s, $-CH_3$); ^{13}C NMR (100 MHz, $CDCl_3$) δ: 145.70, 140.07, 131.17, 126.14, 105.12, 0.09; HRMS(EI) *m/z*: [M+] calcd for $C_{18}H_{20}Br_2S_4Si_2$, 577.8353; found, 577.8347.

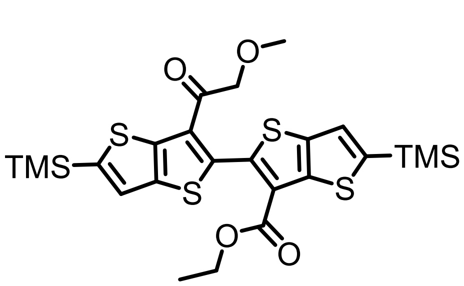

Diethyl-5,5'-bis(trimethylsilyl)-[2,2']-bi-[thieno[3,2-b]thiophenyl]-3,3'-dicarboxylate (**3**)

n-Butyllithium (74.8 mL of a 1.6 M solution in hexanes, 119.7 mmol) was added dropwise to a stirred solution of **2** (17.4 g, 29.9 mmol) in anhydrous diethyl ether (1.2 L) at −90 °C, under an argon atmosphere. The reaction mixture was stirred for 30 min before a solution of ethyl

chloroformate (12.8 mL, 134.2 mmol) in anhydrous diethyl ether (15 mL) was added in one portion. Once the addition was complete, the reaction mixture was allowed to warm to room temperature over 16 h and quenched with water. The crude product was extracted with hexane (500 mL), washed with water (3 × 500 mL), brine (500 mL), dried ($MgSO_4$), and the volatiles removed *in vacuo*. Purification of the crude product by column chromatography over silica (eluent:chloroform) gave a brown oil (12.7 g, 22.5 mmol, 75%). ^{1}H NMR (400 MHz, $CDCl_3$) δ: 7.36 (2H, s, ArH), 4.25 (4H, q, *J* 7.2, -CH2-), 1.20 (6H, t, *J* 7.2, -CH_3), 0.40(18H, s, -CH3); ^{13}CNMR (100 MHz, $CDCl_3$) δ: 161.88, 146.13, 144.93, 142.20, 139.17, 124.70, 124.13, 61.01, 14.07, -0.12; HRMS (ESI) *m/z*: $[M+H]^+$ calcd for $C_{24}H_{30}O_4S_4Si_2$, 567.0644; found,567.0656.

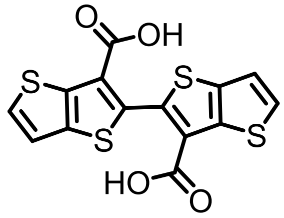

[2,2'-bithieno[3,2-b]thiophene]-3,3'-dicarboxylic acid (**4**)

Sodium hydroxide (3.0 g, 75.0 mmol) was added to a stirred solution of **3** (4.2 g, 7.5 mmol) in methanol (100 mL) and tetrahydrofuran (100 mL), and heated to reflux for 16 h. The reaction mixture was then cooled to 0 ° C before the volatiles were removed *in vacuo*. Concentrated hydrochloric acid (400 mL of a 2M solution) was added, and the resulting precipitate was collected and dried under high vacuum to yield a yellow solid (2.4 g, 6.6 mmol, 88%). ^{1}H NMR (400 MHz, DMSO-d6) δ: 7.82 (2H, d, *J* 5.2, ArH), 7.51 (2H, d, *J* 5.2, ArH); ^{13}C NMR (100 MHz, DMSO-d6) δ:162.81, 140.38, 139.72, 137.07, 130.49, 125.15, 119.73; HRMS (ESI) *m/z*: $[M+H]^+$ calcd for $C_{14}H_6O_4S_4$, 366.9227; found, 366.9240.

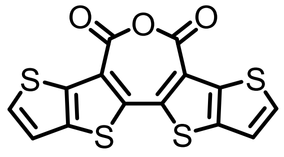

Bisthieno[2',3':4,5]thieno[3,2-c:2',3'-e]oxepine-4,6-dione (**5**)

4 was refluxed in acetic anhydride (15 mL) for 16 h. The reaction mixture was then cooled to 0 °C, filtered and dried under high vacuum, to yield the title compound as a brown solid (212.0 mg, 0.6 mmol, 79%). ^{1}H NMR (400 MHz, DMSO-d6) δ: 7.96 (2H, d, *J* 5.2, ArH), 7.59 (2H, d, *J* 5.2, ArH); ^{13}C NMR (100 MHz, DMSO-d6) δ: 141.88, 141.43, 135.26, 133.22-132.91 (overlapping C), 120.58-120.21 (overlapping C); MS (MALDI-ToF) *m/z*: $[M+H]^+$ 348.8 (40%); Anal. calcd for $C_{14}H_4O_3S_4$: C, 48.26; H, 1.16; found: C, 46.59; H, 0.99.

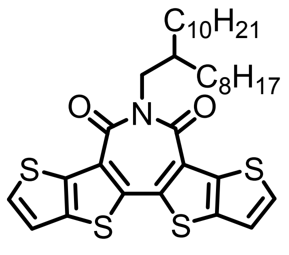

5-(2-octyldodecyl)-4H-bisthieno[2',3':4,5]thieno[3,2-c:2',3'-e]azepine-4,6(5H)-dione (**6**)

5 and 4-(dimethylamino)pyridine (14.0 mg, 0.1 mmol) were added to an oven dried microwave vial equipped with a stirrer bar. The vial was purged with argon before anhydrous toluene (9 mL) was added. A solution of 2-octyldodecyl-1-amine (98.4 mg, 0.3 mmol) in anhydrous toluene (2 mL) was then added dropwise and the reaction mixture was subjected to the following microwave-heating profile: 100 °C for 2 min, 140 °C for 2 min, 180 °C for 2 min and 200 °C for 120 min. Note 9 microwave vials were run sequentially and combined for work-up. After cooling, the crude product was extracted with diethyl ether (150 mL), washed with water (3 × 150 mL), brine (150 mL), dried ($MgSO_4$) and the volatiles removed *in vacuo*, to yield a brown solid (618.6 mg, 1.0 mmol, 18% based upon 1.9 g of total starting material). ^{1}HNMR (400 MHz, $CDCl_3$) δ: 7.61 (2H, d, *J* 5.2, ArH), 7.28 (2H, d, *J* 5.2, ArH), 4.42 (2H, d, *J* 7.2, $-CH_2N-$), 2.05 (1H, m, -CH-), 1.25 (32H, m, $-CH_2-$), 0.86 (6H, m, $-CH_3$); ^{13}C NMR (100 MHz, $CDCl_3$) δ: 160.77, 143.33, 140.19, 134.37, 132.39, 125.31, 118.61, 49.43, 36.17, 32.15-31.98 (overlapping C), 31.63, 30.26, 29.90-29.62 (overlapping C), 29.56-29.43 (overlapping C), 26.49, 22.89-22.77 (overlapping C), 14.29; Anal. calcd for $C_{34}H_{45}NO_2S_4$: C, 65.03; H, 7.22; N, 2.23; found: C, 64.73; H, 7.86; N, 2.28.

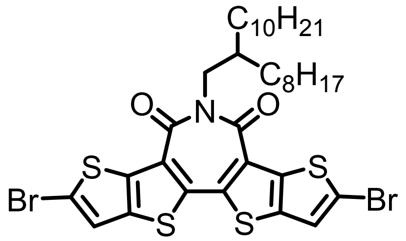

2,8-Dibromo-5-(2-octyldodecyl)-4H-bisthieno[2',3':4,5]thieno[3,2-c:2',3'-e]azepine-4,6(5H)-dione (**7**)

N-Bromosuccinimide (137.4 mg, 0.8 mmol) was added in several portions to a stirred solution of 6 (232.4 mg, 0.4 mmol) and potassium acetate (107.70 mg, 1.1 mmol) in chloroform (25 mL) and acetic acid (25 mL), at room temperature in the absence of light. The reaction was monitored by TLC and upon completion, an aqueous solution of sodium sulfite (50 mL) was added. The crude product was then extracted with chloroform (50 mL) and the combined organics were neutralized with aqueous sodium hydrogen carbonate solution, washed with water (3 × 50 mL), brine (50 mL), dried ($MgSO_4$), and the volatiles removed *in vacuo*. Purification of the crude product by column chromatography over silica (eluent: dichloromethane:hexane, 1:1, (v:v)) gave a yellow solid (112.1 mg, 0.1 mmol, 39%). ^{1}H NMR (400 MHz, TCE-d2) δ: 7.22 (2H, s, ArH), 4.31 (2H, d, *J* 7.2, $-CH_2N-$), 1.97 (1H, m, -CH-), 1.37 (2H, m, -CH2-), 1.22 (30H, s, $-CH_2-$), 0.87 (6H, m, $-CH_3-$); ^{13}C NMR (100 MHz, TCE-d2) δ: 159.84, 142.95, 138.17, 132.80, 124.75, 120.97, 119.60, 49.01, 35.83, 31.90-31.69 (overlapping C), 31.32, 30.02, 29.72-29.44 (overlapping C), 29.27, 26.16, 22.63, 14.17; MS (MALDI-ToF) *m/z*: $[M+H]^+$ 784.2 (95%).

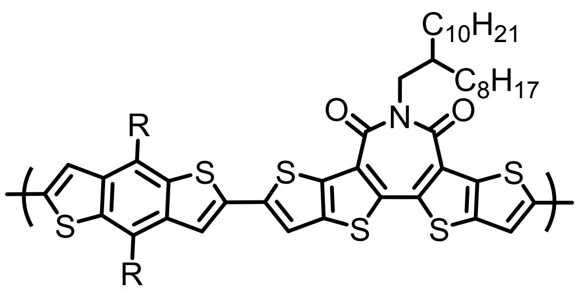

General synthesis procedures for BTTA Polymers (**pBDT-BTTA-1-3**)

7 (1 e.q.), BDT-Tin (1 e.q.), tris(dibenzylideneacetone)dipalladium(0) (0.02 e.q.) and tri(o-tolyl)phosphine (0.08 e.q.) were added to an oven dried microwave vial equipped with a stirrer bar. The vial was purged with argon before anhydrous chlorobenzene (0.8 mL) was added. The reaction mixture was purged with argon for a further 30 min and then subjected to the following microwave-heating profile: 100 °C for 2 min, 120 °C for 2 min, 140 °C for 2 min, 160 °C for 2 min, 180 °C for 20 min, and 200 °C for 20 min. Once the reaction mixture had cooled, the crude product was precipitated in methanol, filtered through a Soxhlet thimble, and purified by Soxhlet extraction: methanol (24 h), acetone (24 h), hexane (24 h) and chloroform (12 h). The chloroform extract was concentrated under vacuum and precipitated in methanol. The resulting precipitate was isolated by filtration and dried under high vacuum, to yield a dark solid.

pBDT-BTTA-1: Yield 60%. Mn: 9 kDa, Mw: 26 kDa, Đ: 2.8. Anal. calcd for $(C_{68}H_{95}NO_4S_6)_n$: C, 69.05; H, 8.10; N, 1.18; found: C, 68.94; H, 8.21; N, 1.29.

pBDT-BTTA-2: Yield 54%. Mn: 50 kDa, Mw: 163 kDa, Đ: 3.3. ^{1}H NMR (500 MHz, TCE-d2) δ: 7.63-7.20 (br, ArH), 7.20-6.71 (br, ArH), 4.58-4.27 (br, $-CH_2N-$), 3.23-2.88 (br, $-CH_2-$), 2.26-0.66 (br, $-CH_3$, -CH2- and -CH); Anal. calcd for $(C_{68}H_{83}NO_2S_8)_n$: C, 67.90; H, 6.96; N, 1.16; found: C, 67.66; H, 7.09; N, 1.25.

pBDT-BTTA-3: Yield 70%. Mn: 42 kDa, Mw: 172 kDa, Đ: 4.1. ^{1}H NMR (500 MHz, TCE-d2) δ: 7.80-6.86 (br, ArH), 4.61-4.33 (br, $-CH_2N-$), 3.22-2.67 (m, -CH2-), 2.20-0.77 (br, -CH3, -CH2- and -CH); Anal. calcd for $(C_{76}H_{99}NO_2S_8)_n$: C, 69.41; H, 7.59; N, 1.07; found: C, 69.24; H, 7.57; N, 1.14.

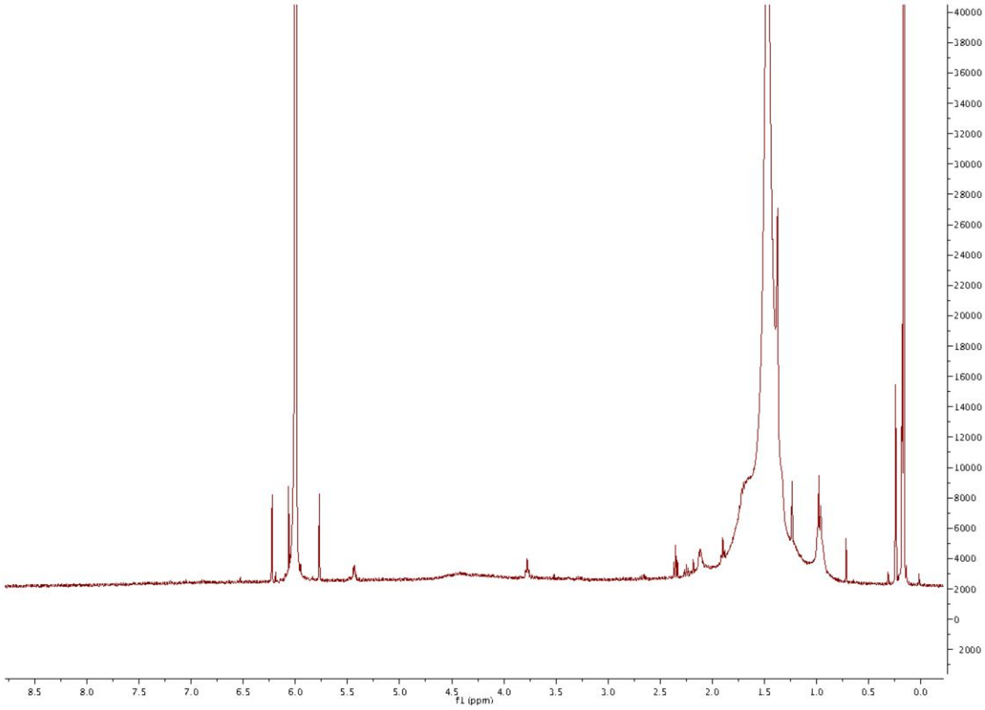

Figure S1. ^{1}H NMR of **pBDT-BTTA-1** at 130 °C in TCE-d2.

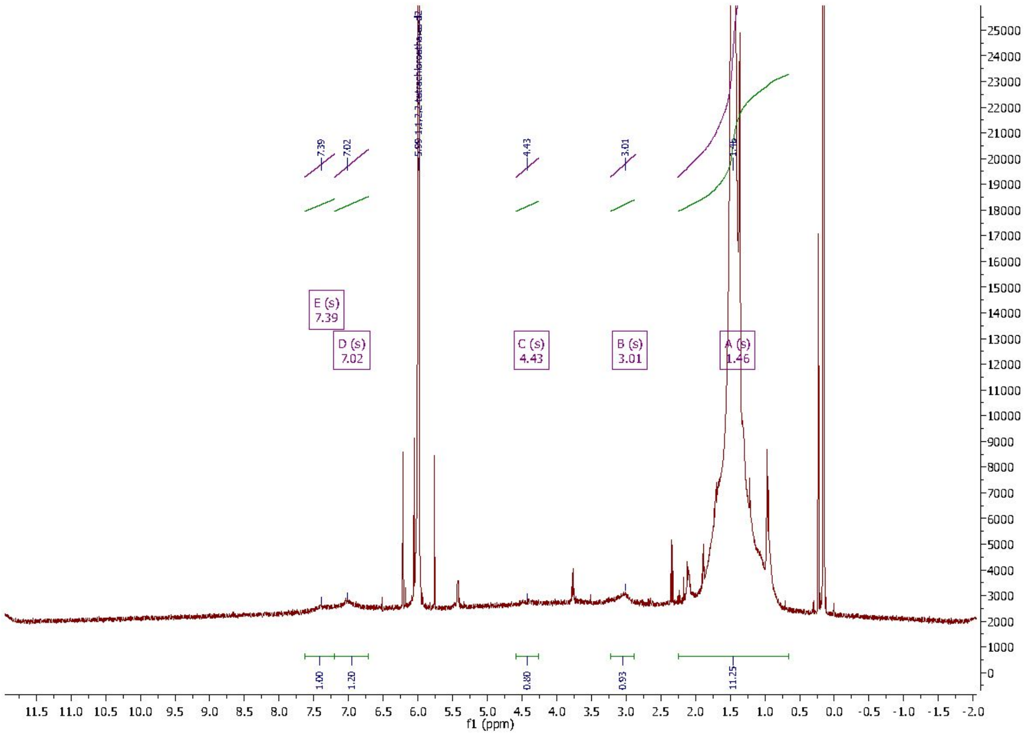

Figure S2. ^{1}H NMR of **pBDT-BTTA-2** at 130 °C in TCE-d2.

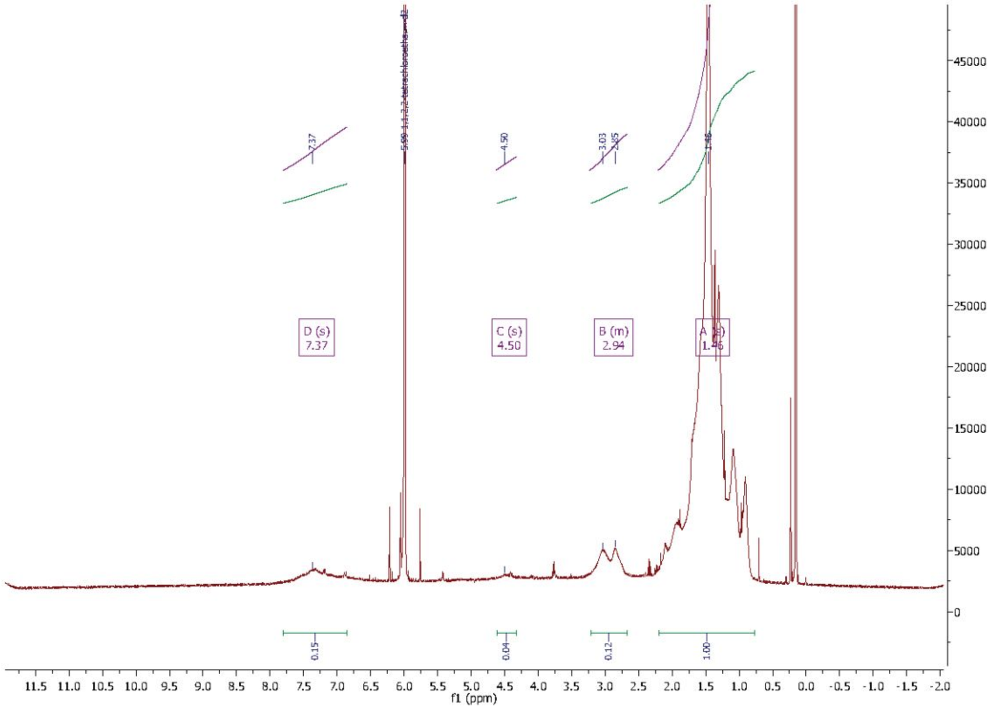

Figure S3. ^{1}H NMR of **pBDT-BTTA-3** at 130 °C in TCE-d2.

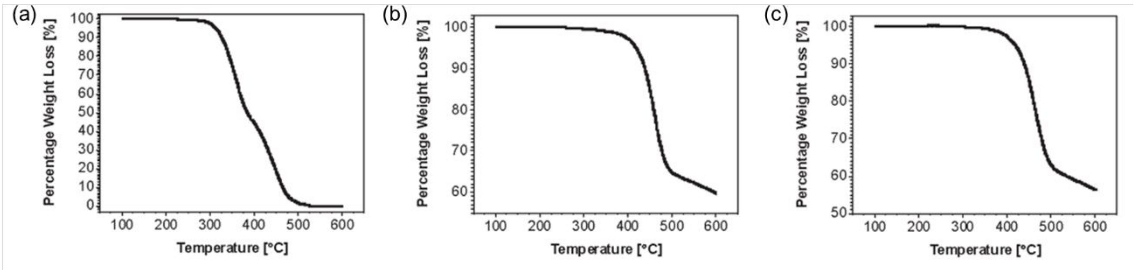

Figure S4. TGA thermograms of a) **pBDT-BTTA-1**, b) **pBDT-BTTA-2**, and c) **pBDT-BTTA-3**, heating at a scan rate of 10 °C min^{-1} under a N_2 atmosphere.

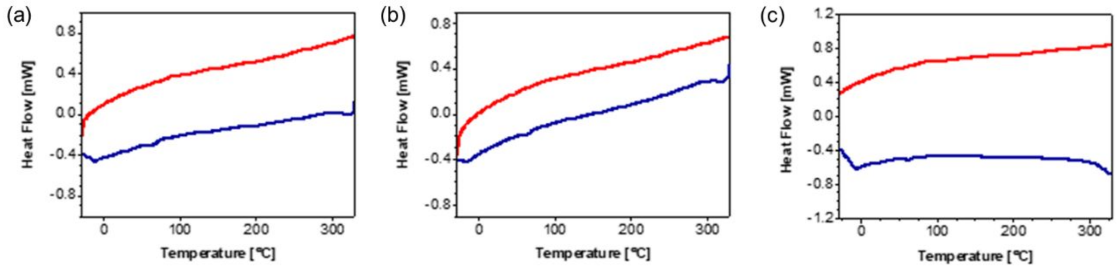

Figure S5. Second heating (red) and cooling (blue) scans of a) **pBDT-BTTA-1**, b) **pBDT-BTTA-2**, and c) **pBDT-BTTA-3** measured by DSC, at a rate of 10 °C min^{-1} under a N_2 atmosphere.

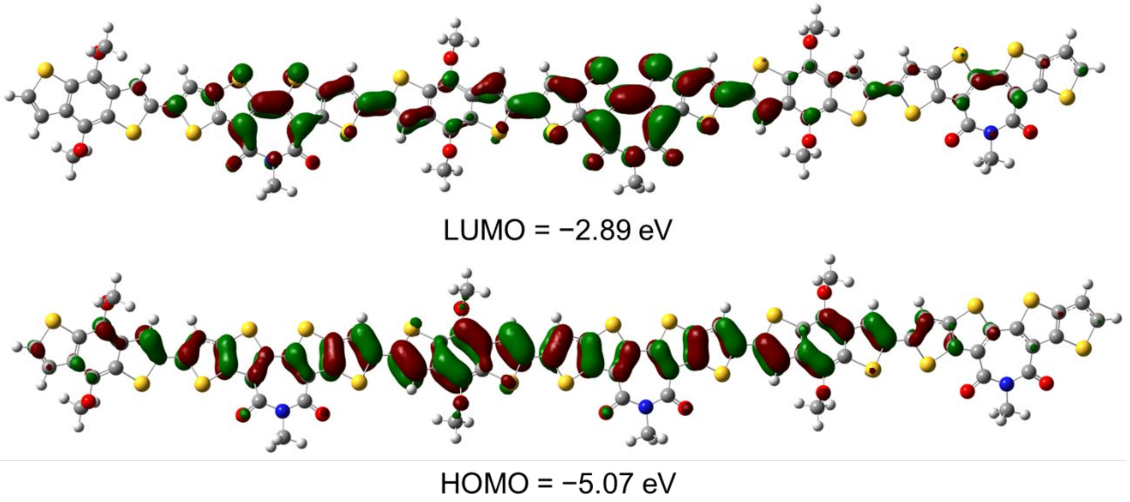

Figure S6. HOMO and LUMO distributions of **pBDT-BTTA-1**.

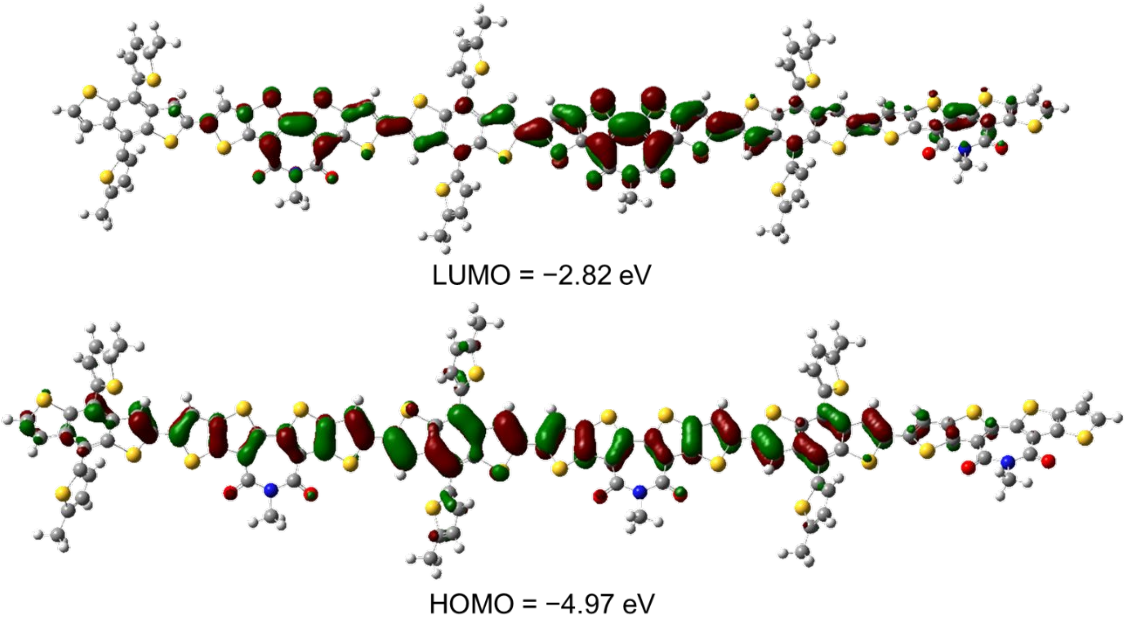

Figure S7. HOMO and LUMO distributions of **pBDT-BTTA-2**.

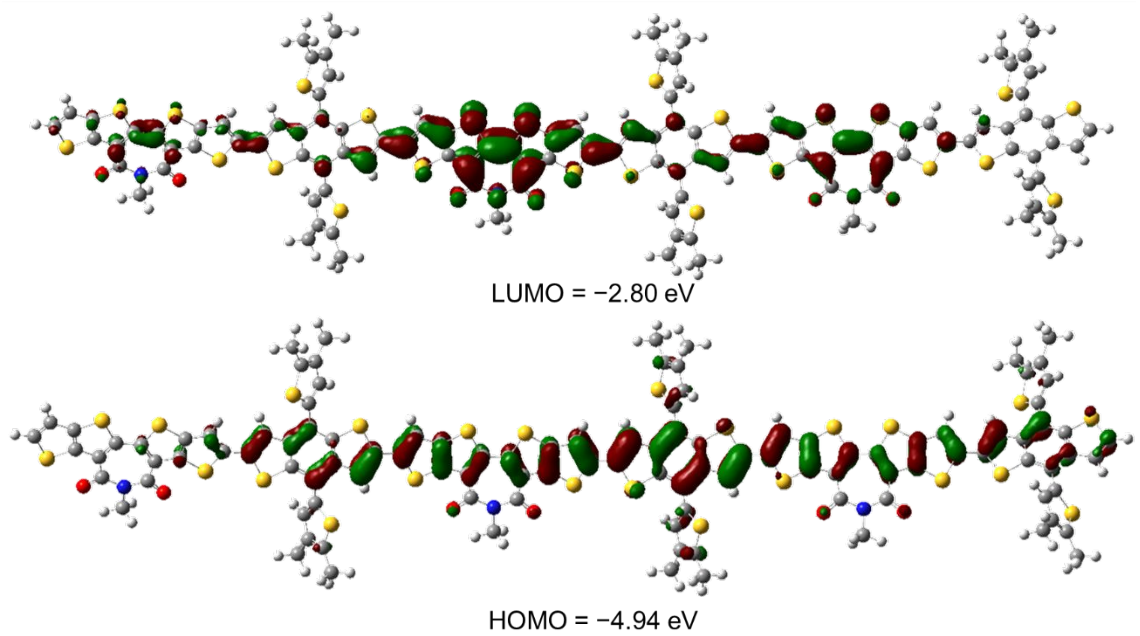

Figure S8. HOMO and LUMO distributions of **pBDT-BTTA-3**.

Table S1. Photovoltaic parameters of pBDT-BTTA-1-3:PC$_{71}$BM-based OPV. The statistics were at least from 10 cells

active layer[a]	solvent	J_{SC} [mA cm^{-2}]	V_{OC} [V]	FF	PCE [%]
**pBDT-BTTA-1**: PC$_{71}$BM	*o*-DCB	–	–	–	–
	CF	7.88 ± 0.13 (7.99)	0.67 ± 0.13 (0.73)	0.44 ± 0.08 (0.47)	2.37 ± 0.76 (2.75)
	CF + 2% DIO	7.13 ± 0.41 (7.67)	0.73 ± 0.01 (0.73)	0.44 ± 0.01 (0.44)	2.31 ± 0.11 (2.44)
**pBDT-BTTA-2**: PC$_{71}$BM	CB	12.90 ± 0.50 (13.58)	0.78 ± 0.03 (0.79)	0.55 ± 0.04 (0.56)	5.58 ± 0.64 (6.09)
	CB + 2% DIO	8.63 ± 0.63 (9.15)	0.65 ± 0.19 (0.75)	0.37 ± 0.04 (0.39)	2.12 ± 0.73 (2.64)
	CB + 2% CN	13.23 ± 0.38 (13.42)	0.80 ± 0.01 (0.81)	0.48 ± 0.06 (0.51)	5.11 ± 0.67 (5.54)
	o-DCB[b]	15.06 ± 1.29 (16.40)	0.64 ± 0.02 (0.65)	0.52 ± 0.06 (0.55)	5.02 ± 0.99 (5.84)
	CF	5.35 ± 0.67 (6.51)	0.85 ± 0.01 (0.85)	0.42 ± 0.01 (0.42)	1.91 ± 0.24 (2.34)
	CF + 2% DIO[c]	5.45 ± 0.65 (5.79)	0.64 ± 0.19 (0.77)	0.38 ± 0.06 (0.36)	1.32 ± 0.42 (1.59)
	CB + 2% CN[c]	8.59 ± 0.74 (9.63)	0.79 ± 0.08 (0.82)	0.40 ± 0.03 (0.41)	2.69 ± 0.44 (3.28)
**pBDT-BTTA-3**: PC$_{71}$BM	CB	9.50 ± 0.20 (9.66)	0.85 ± 0.01 (0.86)	0.64 ± 0.03 (0.66)	5.17 ± 0.27 (5.51)
	CB + 2% DIO	11.30 ± 0.48 (12.20)	0.86 ± 0.02 (0.86)	0.59 ± 0.03 (0.64)	5.80 ± 0.58 (6.70)
	CB + 2% CN	11.40 ± 0.50 (12.10)	0.86 ± 0.06 (0.86)	0.64 ± 0.02 (0.65)	6.27 ± 0.38 (6.78)
	CB + 2% DPE	11.70 ± 0.90 (13.20)	0.86 ± 0.08 (0.87)	0.60 ± 0.01 (0.58)	6.11 ± 0.45 (6.70)

[a]Polymer active layers were spin-coated at 3000 rpm with the exception of [b] which was spin-coated at 1000 rpm and [c] 2000 rpm, from the respective solution (24mg mL^{-1}) in a blend ratio of 1:2 (w:w).

Table S2. Photovoltaic parameters of pBDT-BTTA-3:BTP-eC9 based OPV. The statistics were at least from 10 cells

solvent	annealing [°C]	J_{SC} [mA cm^{-2}]	V_{OC} [V]	FF [%]	PCE [%]
CF	NA	22.60 ± 1.0	0.82 ± 0.004	65.9 ± 0.8	12.2 ± 0.5 (12.8)
	100	23.90 ± 0.9	0.80 ± 0.004	65.1 ± 0.5	12.4 ± 0.4 (12.9)
	150	24.50 ± 0.2	0.76 ± 0.005	62.4 ± 0.6	11.6 ± 0.2 (11.8)
CF + 0.5% DIO	NA	22.20 ± 0.3	0.83 ± 0.008	64.2 ± 1.0	12.0 ± 0.5 (12.5)
	100	22.30 ± 0.1	0.83 ± 0.004	63.9 ± 0.7	11.9 ± 0.1 (12.0)

	150	23.20 ± 0.2	0.81 ± 0.006	58.4 ± 1.3	10.9 ± 0.4 (11.2)
CF + 0.5% CN	NA	24.20 ± 0.5	0.82 ± 0.009	65.6 ± 2.0	13.2 ± 0.3 (13.5)
	100	25.30 ± 0.2	0.79 ± 0.007	65.5 ± 1.0	13.1 ± 0.2 (13.3)
	150	25.90 ± 0.2	0.75 ± 0.013	61.2 ± 2.9	11.8 ± 0.6 (12.5)

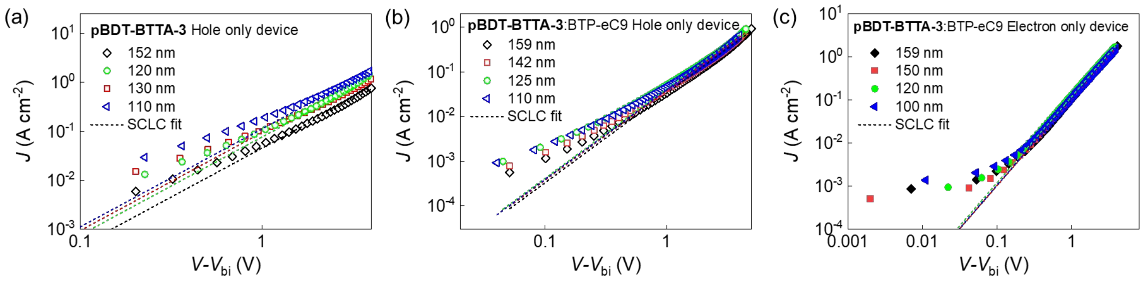

Figure S9. Hole and electron mobilities of **pBDT-BTTA-3** and **pBDT-BTTA-3**:BTP-eC9 blend

Table S3. Hole and electron mobilities of pBDT-BTTA-3 and pBDT-BTTA-3:BTP-eC9 blend

active layer	device type	thickness (nm)	mobility (cm^2 V^{-1} s^{-1})
**pBDT-BTTA-3**	hole	110	(4.60 ± 0.61) × 10^{-4}
		120	(4.77 ± 0.56) × 10^{-4}
		130	(4.91 ± 1.16) × 10^{-4}
		152	(5.17 ± 0.97) × 10^{-4}
**pBDT-BTTA-3:**BTP-eC9	hole	110	(1.75 ± 0.38) × 10^{-4}
		125	(2.45 ± 0.84) × 10^{-4}
		142	(3.65 ± 1.30) × 10^{-4}
		159	(3.98 ± 1.09) × 10^{-4}
**pBDT-BTTA-3:**BTP-eC9	electron	100	(0.36 ± 0.04) × 10^{-3}
		120	(0.63 ± 0.11) × 10^{-3}
		150	(1.10 ± 0.21) × 10^{-3}
		159	(1.32 ± 0.13) × 10^{-3}

References

(1) Becke, A. D. Density-Functional Thermochemistry. Iii. The Role of Exact Exchange. *J. Chem. Phys.* **1993**, *98* (7), 5648-5652.
(2) Lee, C.; Yang, W.; Parr, R. G. Development of the Colle-Salvetti Correlation-Energy Formula into a Functional of the Electron Density. *Phys. Rev. B* **1988**, *37* (2), 785-789.
(3) Kirby, N. M.; Mudie, S. T.; Hawley, A. M.; Cookson, D. J.; Mertens, H. D. T.; Cowieson, N.; Samardzic-Boban, V. A Low-Background-Intensity Focusing Small-Angle X-Ray Scattering Undulator Beamline. *J. Appl. Cryst.* **2013**, *46* (6), 1670-1680.
(4) Danks, A. E.; Hall, S. R.; Schnepp, Z. The Evolution of 'Sol–Gel' Chemistry as a Technique for Materials Synthesis. *Mater. Horiz.* **2016**, *3* (2), 91-112.
(5) Znaidi, L. Sol–Gel-Deposited Zno Thin Films: A Review. *Mater. Sci. Eng. B* **2010**, *174* (1), 18-30.

(6) Liu, S.; Firdaus, Y.; Thomas, S.; Kan, Z.; Cruciani, F.; Lopatin, S.; Bredas, J.-L.; Beaujuge, P. M. Isoindigo-3,4-Difluorothiophene Polymer Acceptors Yield "All-Polymer" Bulk-Heterojunction Solar Cells with over 7 % Efficiency. *Angew. Chem. Int. Ed.* **2018**, *57* (2), 531-535.
(7) Murgatroyd, P. N. Theory of Space-Charge-Limited Current Enhanced by Frenkel Effect. *J. Phys. D: Appl. Phys.* **1970**, *3* (2), 151.
(8) Zhong, H.; Li, Z.; Deledalle, F.; Fregoso, E. C.; Shahid, M.; Fei, Z.; Nielsen, C. B.; Yaacobi-Gross, N.; Rossbauer, S.; Anthopoulos, T. D.; et al. Fused Dithienogermolodithiophene Low Band Gap Polymers for High-Performance Organic Solar Cells without Processing Additives. *J. Am. Chem. Soc.* **2013**, *135* (6), 2040-2043.
